# The Case of Mrs. Catherine Cumming

**Published:** 1852-04-01

**Authors:** 


### THE CASE OF MRS. CATHERINE CUMMING.

---

We append to this number of our Journal an elaborate, and we may add, faithful report (from the shorthand writer's notes) of perhaps one of the most important lunacy cases which has been made the subject of judicial investigation in this country for the last fifty years. We have been anxious to publish a verbatim report of this Commission of Lunacy, not only on account of the deep interest, both public and professional, attached to the case, but in consequence of the many important medico-legal points involved in the inquiry. As this trial has attracted the attention of the legislature, and is likely to give origin to some important modifications of the law, we consider it our duty to place the general and professional evidence on both sides at once before our readers, in order that they may be in a position to form their own unbiassed and unfettered judgment as to its merits. As the question of the right of "traverse" is still *sub judice*, we purposely abstain from making any comments upon the evidence adduced and facts disclosed during the inquiry. The subject will be considered in all its details in our next number. We present this trial, extending over eleven sheets, to our readers without any additional charge. To accomplish this, we have reluctantly been compelled to put aside several valuable articles of great practical interest, all of which will appear in our July number, with copious analyses of several English and foreign works, including both the German, American, and French journals of medical psychology.

